# Adjuvant Hysterectomy in Patients With Residual Disease After Radiation for Locally Advanced Cervical Cancer: A Prospective Longitudinal Study

**DOI:** 10.1200/JGO.18.00157

**Published:** 2019-02-01

**Authors:** Shahana Pervin, Farzana Islam Ruma, Khadija Rahman, Jannatul Ferdous, Rifat Ara, Mollah Mohamed Abu Syed, Annekathryn Goodman

**Affiliations:** ^1^ National Institute of Cancer Research and Hospital, Dhaka, Bangladesh; ^2^ Railway General Hospital, Komlapur, Dhaka, Bangladesh; ^3^ Bangabandhu Sheikh-Mujib Medical University, Dhaka, Bangladesh; ^4^ Massachusetts General Hospital, Boston, MA

## Abstract

**PURPOSE:**

The aim of the study was to evaluate the efficacy of hysterectomy in the control of pelvic disease in patients with post-irradiated residual cervical cancer.

**PATIENTS AND METHODS:**

Forty patients were treated at either National Institute of Cancer Research and Hospital (NICRH) or Delta Cancer Hospital in Dhaka, Bangladesh, with International Federation of Gynecology and Obstetrics stage IIB to IIIB disease with residual disease after the following: either concurrent chemoradiation with or without brachytherapy, induction chemotherapy and external-beam radiotherapy (EBRT) with or without brachytherapy, or only EBRT. Patients were treated by either radical hysterectomy or extrafascial hysterectomy.

**RESULTS:**

From 2009 to June 2013, 55 patients were evaluated for central residual disease on their presentations to NICRH or Delta Hospital. Patients with distant recurrences after primary radiation were excluded. Forty patients had invasive cancer on biopsy and underwent either radical hysterectomy or extrafascial hysterectomy. Surgery was performed 14 to 18 weeks after the initial treatment. Of the 29 women who underwent extrafascial hysterectomy, four (13.8%) developed recurrent disease, and one died; none of the 11 patients treated by radical hysterectomy experienced recurrences during the study period. Morbidity was increased in patients who underwent radical hysterectomy. Overall 90% of patients (36 of 40 patients) who underwent surgery had no evidence of disease at 5 years of follow-up.

**CONCLUSION:**

Surgery is a viable treatment option for patients with residual cervical cancer after radiation. Radical hysterectomy after radiation is more morbid but has better tumor control than extrafascial hysterectomy.

## INTRODUCTION

The World Health Organization estimated 528,000 new cervical cancer occurrences and 266,000 deaths globally each year, which makes it the fourth most common cancer in women.^1^ Nine (87%) of 10 cervical cancer deaths occur in less developed regions.^2^

In Bangladesh, which has a current population of 164,670, cervical cancer is the second leading malignancy and cause of death among Bangladeshi women.^2,3^ There is scant published information about outcomes among those treated for cervical cancer in Bangladesh.^4^ A hospital-based cancer registry was started at National Institute of Cancer Research Hospital (NICRH),the only specialized government tertiary-level cancer hospital in Bangladesh.^5^ NICRH, which has 300 beds, is the biggest cancer hospital in the country.

A notable number of patients with cancer cannot receive immediate radiotherapy as the hospital struggles to cope with the demand for radiotherapy.^6^ On average, 1,000 patients seek appointments for radiotherapy every day at the NICRH. With only six radiotherapy machines, the hospital can offer services to 450 to 500 patients per day. In Bangladesh, cancer mortality is high; 90% of patients present at late stages. Because the majority of the patients in the country seek treatment when their cancer is at an advanced stage, their treatment needs overwhelm the available resources.^5^

At government hospitals, a full course of radiotherapy costs between 15,000 to 30,000 Taka ($176 to $352 US dollars). The majority of patients at NICRH belong to lower and lower-middle income groups and cannot afford the cost of therapy, so they stop halfway through the treatment. Some private hospitals treat patients with cancer who can afford to pay for their health care. Among all private hospitals, two thirds of patients with cancer are treated in Delta private hospital in Dhaka.

The results from large, well-conducted, prospective randomized clinical trials that demonstrated a dramatic improvement in survival when chemotherapy is combined with external-beam radiation therapy (EBRT) and intracavitary radiation therapy (ICRT) have made concurrent cisplatin-based chemotherapy with radiation the standard of care.^7-10^ Treatment of residual cervical disease by hysterectomy after radiation has been used to eliminate potential residual disease for women with locally advanced cervical cancer. A review of 55 women with bulky early-stage cervical cancer who underwent hysterectomies after they received radiation therapy showed a 72.5% survival rate and complication rates similar to controls who had undergone either radiation or radical hysterectomy alone.^11^ The authors concluded that hysterectomy was a viable intervention when there was incomplete treatment response to radiation. For persistent central pelvic disease and central pelvic recurrent disease after radiation therapy, extrafascial hysterectomy, radical hysterectomy, or pelvic exenteration should be considered.^12,13^ This study reports the experience of NICRH and Delta Hospital in Dhaka, Bangladesh, with adjuvant hysterectomy in patients with residual disease after radiation for locally advanced cervical cancer.

## PATIENTS AND METHODS

Data on patients with cervical cancer who both were surgical candidates after radiation and were treated at NICRH and Delta Private Hospital in Dhaka, Bangladesh, was prospectively collected from 2009 to 2013. The hospital ethics committees of both NICRH and Delta approved this study.

Forty patients with locally advanced cervical cancer presented to the NICRH outpatient department, and 15 patients presented to Delta private hospital with symptoms related to persistent cervical cancer after primary treatment of chemotherapy and radiation.

Patients were treated by either linear accelerator or cobalt at NICRH, Delta Hospital, and several other hospitals in Bangladesh with radiation therapy capacity. The ideal and planned dose of radiation was 2 Gy per day for 25 days for a total dose of 50 Gy. Patients who underwent intracavitary implants received three brachytherapy insertions with 7 Gy per insertion.

Twenty-three patients received three doses of single-agent cisplatin to initiate treatment secondary to limited access to radiation therapy resources. International Federation of Gynecology and Obstetrics (FIGO) staging of the original tumors was based on the medical record documentation that the patients brought with them. For eight patients, there was no documentation of a stage assignment, so they were given an unstaged designation. On the basis of physical examination after the primary therapy, these eight women had woody fibrosis in the parametria suggestive of tumor regression, which suggests that their original stages of disease were at least stage IIB. None of these eight women had magnetic resonance imaging (MRI) findings of parametrial involvement.

As a routine practice, all treated patients were encouraged to follow up in the outpatient unit after 6 weeks of completion of radiation and were examined by at least two of the authors in the outpatient department. Examination included a speculum visualization of the cervix, a bimanual examination, and a rectovaginal examination. Patients with examinations suspicious for persistent cancer were advised to return again in 6 weeks. Biopsies were taken in all 55 patients at 12 weeks after completion of treatment. Either computed tomography (CT) scan or MRI was done to exclude parametrial invasion and distant metastasis. The NICRH poor fund was available for patients who were unable to afford radiologic imaging. Other relevant investigations, including chest x-ray and blood work, were also conducted. Patients with suspicious chest findings were also advised to undergo a CT scan of the chest. Fifteen patients (five from Delta Hospital and 10 from NICRH) had distant disease upon evaluation and were excluded from this analysis. Forty patients had biopsy-confirmed persistence of cervical cancer. None of the forty had distant metastasis or parametrial invasion on radiologic imaging. All patients with biopsy-confirmed persistent cervical cancer were given the option to proceed with hysterectomy or to have a nonsurgical option, such as chemotherapy. All of the possible complications were also explained. Patients and their families gave informed consent for hysterectomy. After general conditions were improved, surgery was performed 2 to 6 weeks after biopsy. Both patients with local recurrences and those with distant metastases received carboplatin and paclitaxel chemotherapy.

The decision to perform a radical versus extrafascial hysterectomy was based on surgeon preference and was driven by the surgical findings. For patients in whom there were dense adhesions in the parametria, or when separation of bladder from cervix was difficult because of adhesions, an extrafascial hysterectomy was performed. Each patient was counseled about bladder care, limb massage to prevent lymphedema, and exercise.

Follow-up pelvic assessment was performed every 3 months for 2 years and then every 6 months. Patients who missed any follow-up were called on their cell phones. Vaginal cytology was done every 6 months, and chest x-ray was performed every 3 months for 2 years and then every 6 months. CT scan or MRI was obtained yearly for patients who had pain or bleeding to evaluate for recurrence. After surgery, cervical stromal involvement, surgical margin status, lymph vascular space invasion, and parametrial infiltration were assessed for each pathologic specimen.

## RESULTS

From 2009 through June of 2013, 40 women seen in either the NICRH or the Delta Hospital outpatient department had biopsy-confirmed persistent invasive cervical cancer at least 12 weeks after initial treatment with either radiation or chemoradiation. One third of patients, unable to afford a CT or MRI, were sent to a neighboring government hospital or were supported with the NICRH poor fund resource.

Table 1 lists preoperative characteristics and presurgical therapies. The mean age was 45.1 years. The original assigned FIGO stages were stage IIB for 25 women and stage IIIB for seven women. Eight women, whose diseases were categorized as unstaged, had not been examined by a gynecologic oncologist and had not undergone a standard FIGO staging evaluation before the start of radiation and had at least clinical stage IIB cancers.

**TABLE 1:**
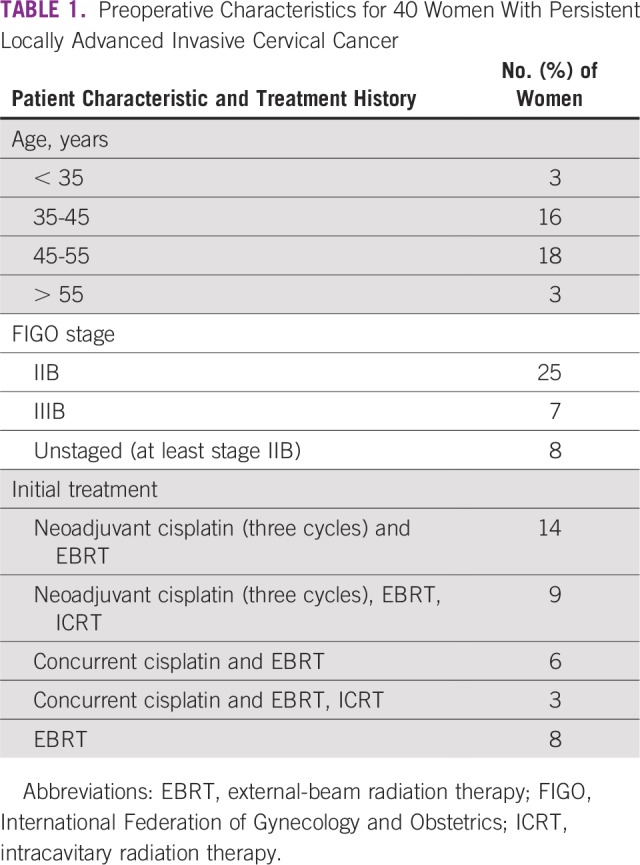
Preoperative Characteristics for 40 Women With Persistent Locally Advanced Invasive Cervical Cancer

Patients had been treated with concurrent chemoradiation (either only EBRT [n = 6 patients] or both EBRT and ICRT [n = 3 patients]) or three cycles of cisplatin followed by either EBRT (n = 14 patients) or both EBRT and ICRT (n = 9 patients). Eight women only received EBRT without chemotherapy or intracavitary radiation.

Preoperatively, 12 women had suspicious cervical tumors greater than 2 cm in size. Twenty-eight women had gross lesions that were less than 2 cm in size. All 40 women had both negative parametrial involvement by examination and MRI or CT scan imaging and negative imaging findings of distant disease.

Table 2 lists the surgical management and outcomes. Twenty-nine women underwent extrafascial hysterectomy and selective lymphadenectomy, of which three women experienced local recurrences and one developed distant metastases. None of the 11 women who underwent radical hysterectomies and complete bilateral lymphadenectomy experienced recurrences. During surgery for both extrafascial hysterectomy and radical hysterectomies, estimated blood loss ranged from 450 mL to 550 mL, and the average duration of surgery was 2.5 hours. There were no surgical or postoperative complications in the extrafascial hysterectomy group. Among the 11 women who underwent radical hysterectomies, one patient had a rectal injury; one had bladder injury; and one experienced a vascular injury; the resulting intraoperative injury rate was 27%. All injuries were repaired successfully. The mean time of hospital stay was 8 days. During the postoperative period, two patients developed urinary retention (grade 1 by common toxicity criteria), and two had ureteral stenosis (grade 3 toxicity), which provided a postoperative adverse event rate of 36%. These postoperative events were treated successfully and resolved. Three patients (27%) had lymphedema (grade 2 toxicity) as late complications in the radical hysterectomy group. There were no late complications among the extrafascial hysterectomy group.

**TABLE 2:**
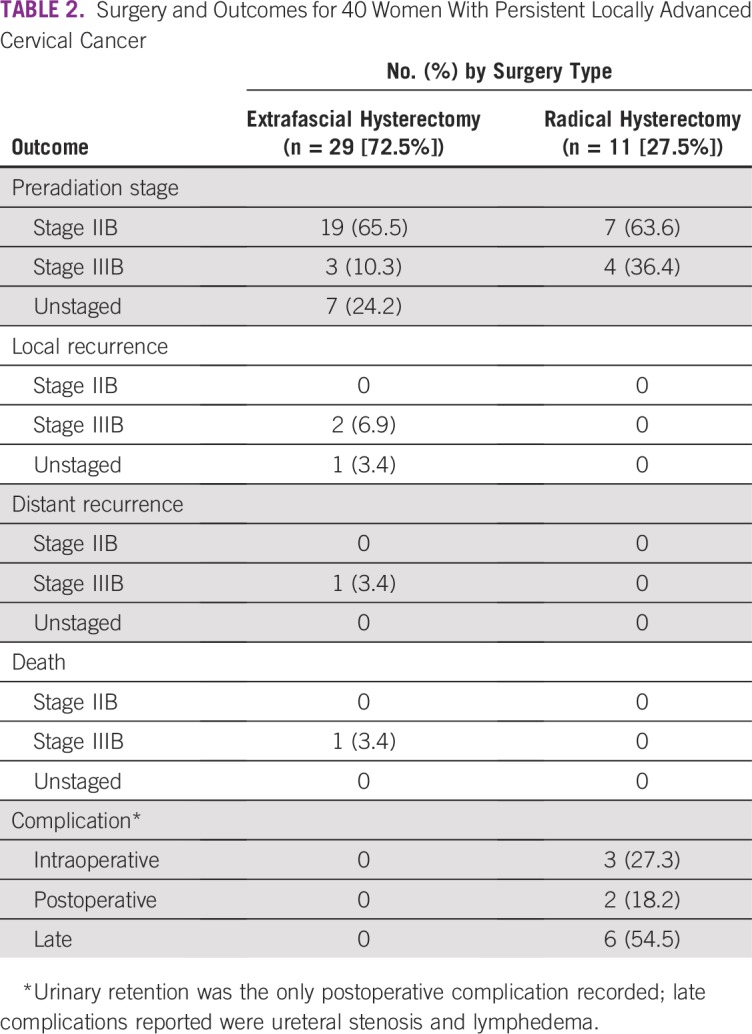
Surgery and Outcomes for 40 Women With Persistent Locally Advanced Cervical Cancer

Pathologic examination (Table 3) of hysterectomy specimens showed deep stromal invasion in eight specimens and parametrial involvement in two patients. There were no positive surgical margins. Although 10 patients had grossly enlarged pelvic nodes, lymph nodes were pathologically negative for tumor. Thirty-two women had squamous cell cancers, seven had adenocarcinomas, and one had an adenosquamous cancer. Pathologic grade was grade 1 in 10 patients, grade 2 in 26 patients, and grade 3 in four patients.

**TABLE 3:**
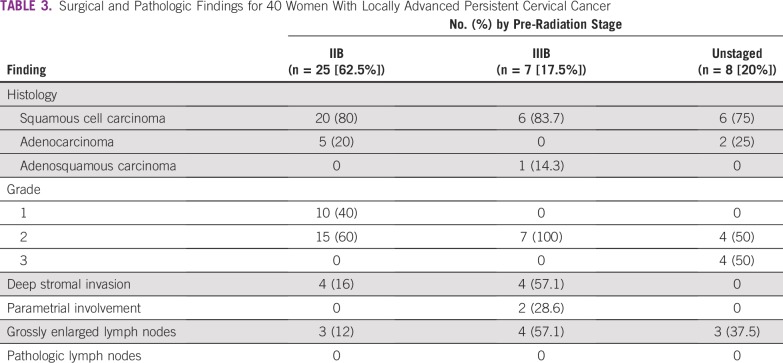
Surgical and Pathologic Findings for 40 Women With Locally Advanced Persistent Cervical Cancer

Patients were observed between 5 and 9 years (mean, 7.8 years). Table 4 lists the number of women treated each year and their follow-up statuses.

**TABLE 4:**
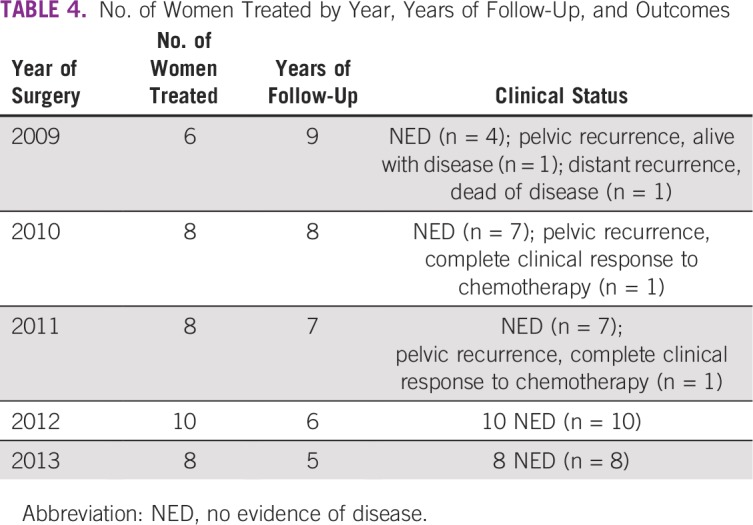
No. of Women Treated by Year, Years of Follow-Up, and Outcomes

Table 5 lists the clinical features and clinical course of the four women with recurrent disease after surgery. All recurrences occurred in the extrafascial hysterectomy group, and no one with a tumor size less than 2 cm experienced recurrence. Three women developed local pelvic recurrences; one was on the pelvic sidewall, and two were at the vaginal cuff. Two of these had a complete clinical response after they received six cycles of carboplatin and paclitaxel and remained disease free at 4 and 5 years of follow-up after chemotherapy, respectively. The third patient also received six cycles of carboplatin and paclitaxel, experienced a disease remission, and then experienced recurrence 12 months later. She is currently alive with disease. One patient with distant metastases in a mediastinal lymph node 32 months after surgery was treated with carboplatin and taxol but experienced progression and died 2.5 years later. There were no recurrences by 5 years of follow-up in the 11 women treated by radical hysterectomy.

**TABLE 5:**
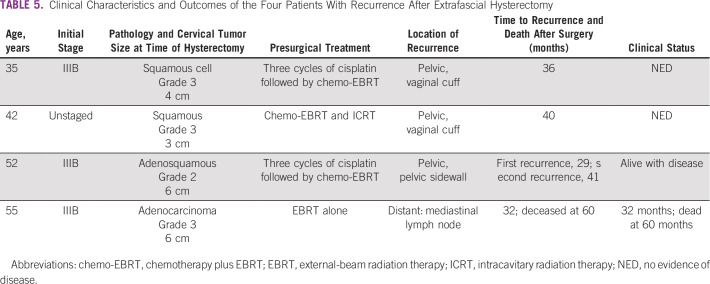
Clinical Characteristics and Outcomes of the Four Patients With Recurrence After Extrafascial Hysterectomy

## DISCUSSION

At NICRH in general, less than 20% of patients are able to follow up after primary treatment. Delta Hospital in contrast has follow-up rates of greater than 80%. Those patients, able to return or come to the outpatient department after treatment, had symptoms and exams suspicious of residual disease. We report on the management and outcomes of disease in 40 post-irradiated patients with cervical cancer who had surgically resectable residual disease.

Multiple challenges to the appropriate treatment and follow-up for women with invasive cervical cancer in Bangladesh include inadequate primary cervical cancer screening that leads to identification of advanced cancers at presentation. Although the female literacy rate has increased from 18% in 1981 to 70% in 2016, there is still little understanding of medical conditions, such as cervical cancer, among the general population.^14,15^ Bangladesh is 139 of 188 countries on the Human Development Index.^16^ The majority of the Bangladeshi population is poor and has no means to pay for health care.^17^

Radiation therapy machines are limited in Bangladesh, and long waiting lists exist to receive therapy; only 11% of patients with cancer complete radiotherapy treatment regimens.^18,19^ As a result, when women in Bangladesh present with advanced cervical cancer, it is common for them to not receive the internationally accepted standard of cancer care.^10^

It is challenging to fully know the extent of the problem of access to appropriate cancer care and the outcomes for women with advanced cervical cancer, because there is currently no national tumor registry in Bangladesh. Also, after treatment, many women either do not understand the importance of follow-up or cannot afford it.

Pelvic radiation therapy alone fails to control locally advanced cervical cancer in 35% to 90% of patients; the ability of radiotherapy alone to cure bulky cervical cancer is limited by the size of the tumor.^20,21^ Residual tumor after primary treatment is the ultimate cause of local recurrences.^22,23^ Even with the delivery of standard-of-care chemoradiation with brachytherapy, 35% of patients experience disease progression.^24^ Other challenges to local tumor control include delays in the start of therapy, inadequate radiation dosing, and lack of the use of brachytherapy.

When adequate radiation is not available, chemotherapy and surgical management have been used for locally advanced cervical cancers. The addition of neoadjuvant chemotherapy both as a delay tactic during the wait for radiation and as a strategy to potentially render unresectable disease resectable has been reported in resource-limited countries.^25,26^ A recent randomized trial to compare neoadjuvant chemotherapy followed by hysterectomy versus adequate chemoradiation for women with stage IB2, IIA, or IIB squamous cell carcinoma of the cervix suggests the superiority of chemoradiation.^27^ The 5-year disease-free survival of women who received neoadjuvant chemotherapy was 69.3% compared with 76.7% for the chemoradiation group (hazard ratio, 1.38). However, the overall 5-year survival for both groups was similar (75.4% *v* 74.7%, respectively, with a hazard ratio of 1.025), but the study was not powered for overall survival.

A second therapeutic strategy to manage treatment for patients who have not received adequate radiation is surgical resection of residual disease. In the setting of adequate EBRT and ICRT for bulky stage IB2 cervical cancers, the addition of extrafascial hysterectomy greatly reduces pelvic relapse but did not improve survival.^28^ Other more recent reports of adjuvant hysterectomy after adequate radical chemoradiotherapy confirm the lack of survival benefit.^29,30^ However, in the setting when adequate chemoradiation is not available, single-institution series suggest that there is a salvage rate to the addition of surgery. Walji et al^31^ documented the role of hysterectomy for unsuccessful brachytherapy in patients with cervical carcinoma. Hysterectomy was performed after unsuccessful brachytherapy in 19 patients, and there were no recurrences during 5 years of follow-up.^31^ In another study, 38 patients underwent extrafascial hysterectomies because of the inability to receive brachytherapy as a result of cervical stenosis.^32^ The overall survival was 71% in a median follow-up period of 3 years. Three patients had vaginal cuff recurrences.

We report our experience with the surgical salvage of disease in 40 women with residual invasive cervical cancer after various combinations of chemotherapy and radiation. All of our patients had histologic evidence of residual cancer in the surgical specimens. Interestingly, none of these patients had microscopic involvement of lymph nodes, which suggests that the primary radiation adequately sterilized any nodal disease. An important prognostic factor was the size of residual disease, and the best outcomes were in patients with a tumor size of less than 2 cm. In our study, none of the patients with a tumor size of less than 2 cm experienced recurrence. In addition, only patients in the group undergoing an extrafascial hysterectomy experienced recurrence, whereas none of those who underwent radical hysterectomies experienced a recurrence. Intraoperative, postoperative, and long-term complications were noted in the radical hysterectomy group.

The strength of this study was the long-term follow-up of a group of women with locally advanced cervical cancer who underwent multimodality therapy. All women underwent surgery by the same group of gynecologic oncology surgeons. There are several limitations in our study. The major challenge to the interpretation of our data are the lack of standardized preoperative therapy. The reasons for the lack of consistent treatment of bulky early-stage disease were multifactorial, and the treating physicians were challenged in understanding the details of the chemotherapy and radiation dosing of these 40 women. We had a limited number of patients: data from many women were lost to follow-up after they received some initial treatment of their advanced cancers. The loss to follow-up of many patients with cervical cancer was due to the extreme poverty of most patients, their inability to pay for treatment, or the lack of available radiotherapy to adequately treat them. Of the patients with residual cancer who did return for follow-up, only three of them had received the internationally accepted standard of concurrent chemotherapy with EBRT followed by intracavitary radiation implants. In addition, we have incomplete records of the radiation dosing on many of our patients. In Bangladesh, there are no centralized medical record keeping in hospitals, so patients and their families carry their records, and many details of care can be lost in the process. Eight of the 40 patients had no pretreatment staging. Another limitation of our study is that the decision to perform an extrafascial versus a radical hysterectomy was individualized by the operating surgeon, and it is hard to extrapolate guidelines for the radicality of surgery on the basis of these data.

In conclusion, surgical intervention by either extrafascial or radical hysterectomy should be strongly considered for women with biopsy-confirmed residual disease after chemoradiation. For general gynecologic surgeons who work in regions where gynecologic oncologists are not available, they should consider performance of an extrafascial hysterectomy for women who have uterus-confined persistent cervical cancer after radiation therapy. The data from this small study has encouraged us to aggressively evaluate patients with potentially inadequate primary therapy and to routinely consider hysterectomy for persistent disease. Given the limitations of our study, a larger prospective trial to evaluate extrafascial versus radical hysterectomy would help guide what should be the standard of care for salvage therapy. Physicians who work in resource-limited regions where women may not receive adequate radiation should have a strong index of suspicion to evaluate women with locally advanced cervical cancer for residual disease. Close clinical follow-up is crucial to identify these women in a timely manner.

## Data Availability

The following represents disclosure information provided by authors of this manuscript. All relationships are considered compensated. Relationships are self-held unless noted. I = Immediate Family Member, Inst = My Institution. Relationships may not relate to the subject matter of this manuscript. For more information about ASCO's conflict of interest policy, please refer to www.asco.org/rwc or ascopubs.org/jco/site/ifc. No potential conflicts of interest were reported.
